# A Young *Drosophila* Duplicate Gene Plays Essential Roles in Spermatogenesis by Regulating Several Y-Linked Male Fertility Genes

**DOI:** 10.1371/journal.pgen.1001255

**Published:** 2010-12-23

**Authors:** Yun Ding, Li Zhao, Shuang Yang, Yu Jiang, Yuan Chen, Ruoping Zhao, Yue Zhang, Guojie Zhang, Yang Dong, Haijing Yu, Qi Zhou, Wen Wang

**Affiliations:** 1 State Key Laboratory of Genetic Resources and Evolution, Kunming Institute of Zoology, Chinese Academy of Sciences, Kunming, China; 2 Graduate University of Chinese Academy of Sciences, Beijing, China; 3 Laboratory for Conservation and Utilization of Bio-Resources and Human Genetics Center of Yunnan University, Kunming, China; University of Michigan, United States of America

## Abstract

Gene duplication is supposed to be the major source for genetic innovations. However, how a new duplicate gene acquires functions by integrating into a pathway and results in adaptively important phenotypes has remained largely unknown. Here, we investigated the biological roles and the underlying molecular mechanism of the young *kep1* gene family in the *Drosophila melanogaster* species subgroup to understand the origin and evolution of new genes with new functions. Sequence and expression analysis demonstrates that one of the new duplicates, *nsr* (*novel spermatogenesis regulator*), exhibits positive selection signals and novel subcellular localization pattern. Targeted mutagenesis and whole-transcriptome sequencing analysis provide evidence that *nsr* is required for male reproduction associated with sperm individualization, coiling, and structural integrity of the sperm axoneme via regulation of several Y chromosome fertility genes post-transcriptionally. The absence of *nsr*-like expression pattern and the presence of the corresponding *cis*-regulatory elements of the parental gene *kep1* in the pre-duplication species *Drosophila yakuba* indicate that *kep1* might not be ancestrally required for male functions and that *nsr* possibly has experienced the neofunctionalization process, facilitated by changes of *trans*-regulatory repertories. These findings not only present a comprehensive picture about the evolution of a new duplicate gene but also show that recently originated duplicate genes can acquire multiple biological roles and establish novel functional pathways by regulating essential genes.

## Author Summary

Gene duplication has long been appreciated as a major source for new genes and new functions. Nevertheless, it is still a fascinating mystery how new duplicate genes are functionally integrated into the existing gene network and how they contribute to the novel functions of organisms at the pathway level. By studying the recently originated *kep1* gene family in *Drosophila melanogaster*, we show that one of the young duplicate genes, *nsr*, has evolved important biological functions associated with male reproduction by regulating several essential fertility genes in the short evolutionary period after its birth. The evolutionary dynamics, biological roles, and the underlying molecular mechanism of *nsr* revealed in this study present a vivid and comprehensive example of how new genes acquire important biological functions and demonstrate that recently originated new genes can regulate pre-existing essential genes and create novel architectures of genetic pathways.

## Introduction

Gene duplication is a fundamental evolutionary process and provides a major source for genetic novelties [1]–[3]. The usual fate of a gene duplicate is pseudogenization, but some duplicates can fortuitously survive through neofunctionalization, in which one copy retains its ancestral function while the other copy acquires a novel function, or subfunctionalization, in which the duplicate and the ancestral copies subdivide the ancestral functions [4], [5]. The two processes, especially neofunctionalization, should have contributed greatly to the biological diversity by providing genetic innovations.

However, how a new duplicate gene acquires functions by integrating into a pathway and results in adaptively important phenotypes has remained largely unknown. Studying the recently originated young genes could be a very informative way to illustrate these processes, as genes at the early stage of evolution should have retained their original features well, which could have changed with time [3]. Currently, a number of young duplicate genes with potential biological functions have been reported [6]–[13]. Among them, three young *Drosophila* duplicate genes, arisen by retroposition, were reported to have male-related functions: *K81* was proposed to be a testes-expressed paternal effect gene [6], *mojoless* is required for male germline survival [7], and *sphinx* is an RNA-coding gene responsible for male courtship behavior [8], [14]. Nevertheless, little is known about how these young duplicate genes have been integrated into the molecular pathways and thereby have realized their functions in the host species.

In this study, we systematically characterized a young *Drosophila* gene of the *kep1* gene family, which originated recently in the *Drosophila melanogaster* (*D. melanogaster*) species complex (including *D. melanogaster*, *D. simulans*, *D. mauritiana,* and *D. sechellia*) about 5.4–12.8 million years ago through the duplications of the *kep1* gene locus, mediated by the transposon DNAREP1_DM [15]. We performed a comprehensive investigation of its functions within an evolutionary context and successfully revealed its biological roles as well as the underlying molecular mechanism. The results shed novel light on the functional origin of new genes at the pathway level.

## Results

### Evolutionary Analysis of the *kep1* Gene Family

**Figure 1:**
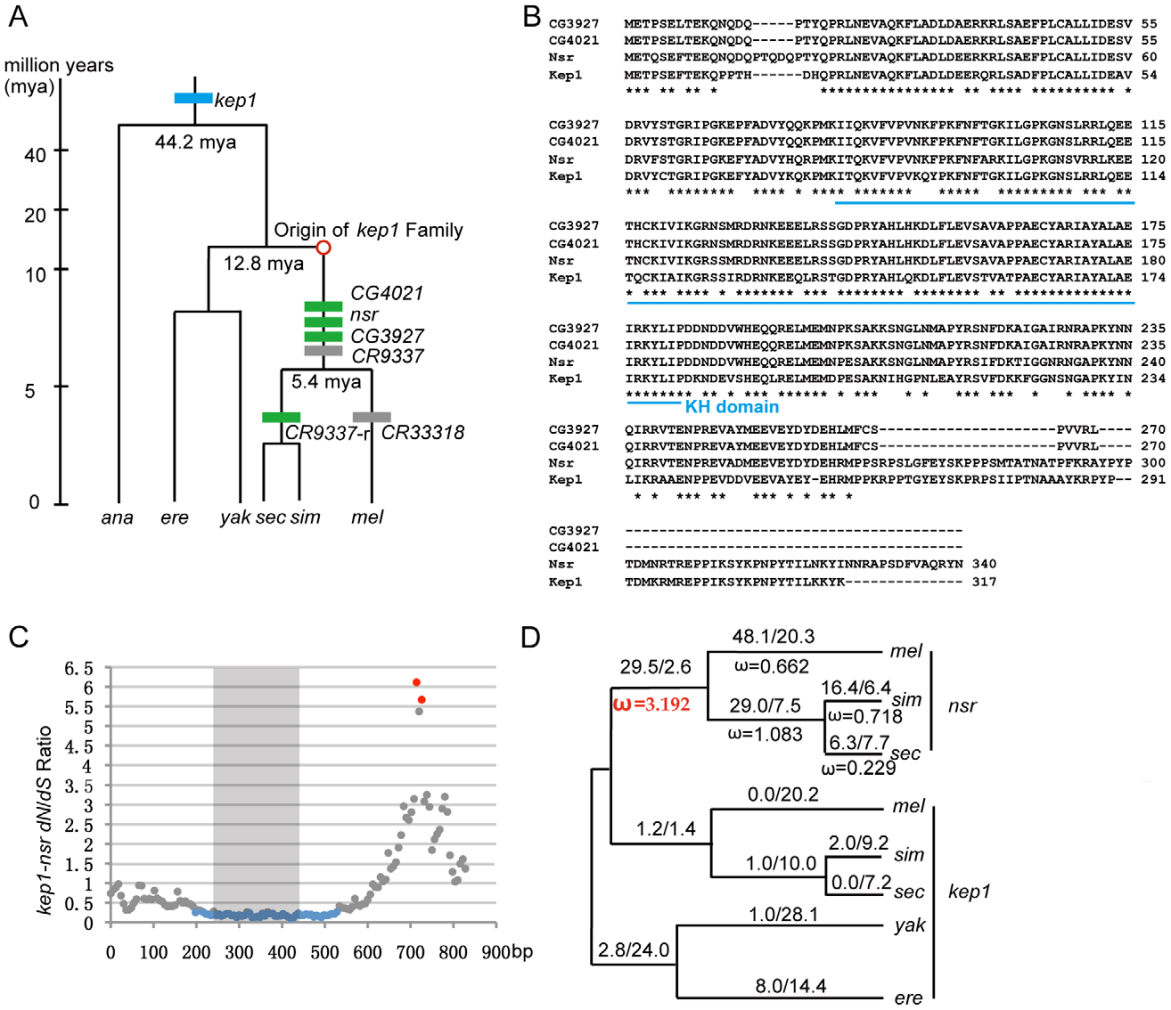
Evolutionary analysis of *kep1* family. (A) Origination events of the newly originated *kep1* gene family. The phylogeny of the *Drosophila* species and the divergence time are indicated [18]. On the phylogenetic tree, the blue box represents the parental gene *kep1*, and green and grey boxes represent the intact new genes and pseudogenes of the *kep1* family, respectively. The approximate starting point of the origination of the *kep1* family is depicted as a red circle. (B) Multiple alignments for protein sequences of *kep1* family genes in *D. melanogaster*. The asterisks denote the positions of identical amino acids. The blue line under the alignment shows the KH RNA-binding domain. (C) Distribution of *dN*/*dS* throughout the *kep1*-*nsr* pair. With 120-bp windows and 6-bp slides, *dN*/*dS* ratios were estimated using the maximum likelihood method [53] and plotted. Blue and red spots represent *dN*/*dS* ratios that are statistically significantly lower and higher than the neutral expectation (*p-*value <0.05, two-tailed Fisher's exact test), respectively. Regions embedded in the KH domain, as depicted by the grey block, are enriched with signals of purifying selection. (D) Likelihood values of nucleotide substitutions for *nsr* in *Drosophila* lineages. Numbers of nonsynonymous and synonymous substitutions for the entire coding sequences are labeled above the lineages, and *ω* values (*dN*/*dS*) are labeled beneath the lineages. A *ω* value representing the lineage that shows significant evidence of positive selection is highlighted in red. Abbreviations: *D. melanogaster* (*mel*); *D. simulans* (*sim*); *D. sechellia* (*sec*); *D. yakuba* (*yak*); *D. erecta* (*ere*), and *D. ananassae* (*ana*).

There are 7 members in the *kep1* gene family, and their phylogenetic distributions are illustrated in Figure 1A. The parental gene *kep1* is present in all *Drosophila* species. Through the duplications of the *kep1* gene locus, the new genes *nsr* (*novel spermatogenesis regulator*, *CG3875*), *CG3927*, *CR9337,* and *CG4021* originated in the common ancestor of the *D. melanogaster* species complex, and *CR9337-r* and *CR33318* occurred after the sibling species in the complex diverged [15]. In this study, we focused on the intact new duplicates *nsr*, *CG3927,* and *CG4021* in *D. melanogaster*, in which the genetic manipulations are feasible.

The *kep1* family copies are located dispersedly on the second chromosome. *D. melanogaster kep1* is a pre-mRNA splicing factor, influencing female fertility, eye development, and immune responses to bacterial infection [16]. Consistent with that, the coding sequences of *kep1* are conserved throughout the *Drosophila* phylogeny (Table S1). Multiple alignments of the protein sequences of *kep1* family members show that the three intact new genes have a well-retained KH RNA-binding domain but possess highly diverged C-termini (Figure 1B). By sliding window analysis, the ratio of nonsynonymous changes (*dN*) over synonymous changes (*dS*) for each *kep1*-new gene pair was estimated and tested for selection. For all gene pairs, significant purifying selection signals are enriched in the KH domain region (Figure 1C and Figure S1A), revealing functional constraint on the new genes. Most interestingly, the C-termini between the *kep1*-*nsr* pair shows significant positive selection signal (*dN*/*dS*  = 6.11, *p*-value <0.05) (Figure 1C), which probably arose from accelerated evolution in the *nsr* as a result of adaptive evolution.

We analyzed the evolutionary patterns along the phylogenetic branches for *nsr* (Figure 1D), *CG3927,* and *CG4021* (Figure S1B), based on the maximum likelihood estimates of *ω* values (*dN*/*dS*) [17]. If we assume that the duplication events happened when *D. melanogaster* and *D. yakuba* diverged 7.4 million years ago [18], even using the most conservative estimate of the synonymous substitution rate for *Drosophila* [19]–[21], 24.3, 17.9, and 22.6 synonymous substitutions are expected to occur in the ancestral lineage of the *D. melanogaster* species complex for *nsr*, *CG3927,* and *CG4021*, respectively. These numbers are far beyond our observations, which are 2.6 for *nsr*, 0 for *CG3927,* and 9.3 for *CG4021* (Figure 1D and Figure S1B). Therefore, the three new duplicate copies must have originated very late in the ancestral lineage, probably close to the split point of the sibling species in the *D. melanogaster* species complex. In the ancestral lineage, there are many nonsynonymous substitutions in the new genes, and the estimated *ω* values are 3.192 for *nsr* (Figure 1D), infinite for *CG3927* (there are no synonymous mutations), and 1.149 for *CG4021* (Figure S1B), in which the ones for *nsr* and *CG3927* are significantly larger than the neutral expectation (Table S2), indicating that positive selection should have shaped the two new genes, especially *nsr*. On the branches leading to individual species, the *ω* values decline, possibly because the new genes might have evolved functions that are under selective constraint.

### Tissue-Specific Expression and Subcellular Localization of New Duplicate Genes in the *kep1* Family

**Figure 2:**
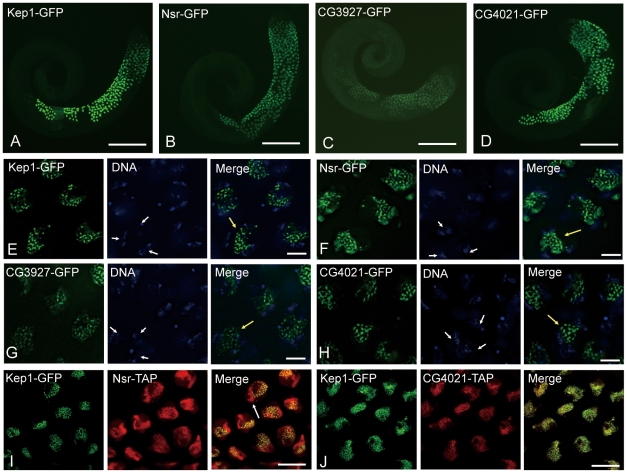
Expression analysis of *kep1* family proteins by GFP transgene in *D. melanogaster*. (A–D) Low-magnification fluorescent images of transgenic GFP-fused Kep1 (A), Nsr (B), CG3927 (C), and CG4021 (D) proteins (green) in testes of *D. melanogaster* strain *w1118*. All of the *kep1* family genes are enriched in the primary spermatocytes. (E–H) High-magnification fluorescent images of transgenic GFP-fused Kep1 (E), Nsr (F), CG3927 (G), and CG4021 (H) proteins in the primary spermatocytes. Proteins of *kep1* family genes are located in the nuclear regions of the primary spermatocytes, which are distinguishable from the three diffusely staining nuclear regions (three white arrows), corresponding to the three main chromosome bivalents. Note that all *kep1* family proteins are distributed in speckles (yellow arrow). (I and J) Comparisons of subcellular localization between Kep1 and new gene proteins. Kep1 is tagged with GFP (green); while Nsr and CG4021 are tagged with TAP (red), respectively. Nsr protein shows a wider expression region than Kep1 protein (arrow) (I), and CG4021 protein is completely co-localized with Kep1 protein (J). Scale bars: 200 µm for A–D; 10 µm for E–H; 50 µm for I and J.

In *D. melanogaster*, the *kep1* copy is ubiquitously expressed [22], but the new duplicate copies display a male-specific expression pattern, according to our RT-PCR results (Figure S2A). To provide clues for the biological functions of new *kep1* family genes, GFP was fused to the coding sequences of each gene to designate their detailed expression patterns in *D. melanogaster* (Figure S2B). Since the uniform male-specific expression pattern for all of the new duplicate genes is more likely a consequence of a shared regulatory region rather than independently evolved genetic mutations, we used the homologous upstream regulatory sequences of all *kep1* family genes as the driving promoter (Figure S2D). As expected, the shared regulatory region is sufficient to drive similar male-specific expression for each of the GFP-tagged *kep1* family proteins, which are unexceptionally enriched in the primary spermatocytes of testes (Figure 2A–2D). Previous large-scale profiling of gene expression patterns in *D. melanogaster* testes demonstrated that all *kep1* family genes showed a high level of mitosis and meiosis expression, followed by much-reduced post-meiosis expression [23]. This result is consistent with our observation and also suggests that the *kep1* family genes may be expressed in the spermatogonial stage as well.

In the primary spermatocytes, *kep1* family proteins are localized in a specked nuclear pattern (Figure 2E–2H), a highly diagnostic feature for spliceosomal components [24], [25]. Considering that *D. melanogaster kep1* is a splicing factor responsible for the alternative splicing of the *Drosophila* caspase molecule *dredd* [16], the observation above led us to speculate that new *kep1* family genes might regulate the pre-mRNA processing of genes required for spermatogenesis and sperm function.

Evolution of novel subcellular localization after duplication is thought to be an important evolutionary mechanism for the origins of genes with novel functions [26]. Though both are distributed in punctuate nuclear structures of primary spermatocytes, the localization of Nsr protein is much broader than the Kep1 protein (Figure 2I). RNase A treatment of testes could lead to the ectopic accumulation and dispersal of GFP-tagged Nsr protein (Figure S2E, S2F, S2G, 2H), indicating that the Nsr protein is localized in an RNA-dependent manner, and its expanded nuclear localization might imply a novel RNA-binding property. CG4021 protein is localized, completely overlapping with the Kep1 protein, in primary spermatocyte nuclei (Figure 2J), and CG3927 protein was found to have a lack of a significant fluorescent signal for the comparison.

### Loss-of-Function Analysis for the *kep1* Family Genes

**Figure 3:**
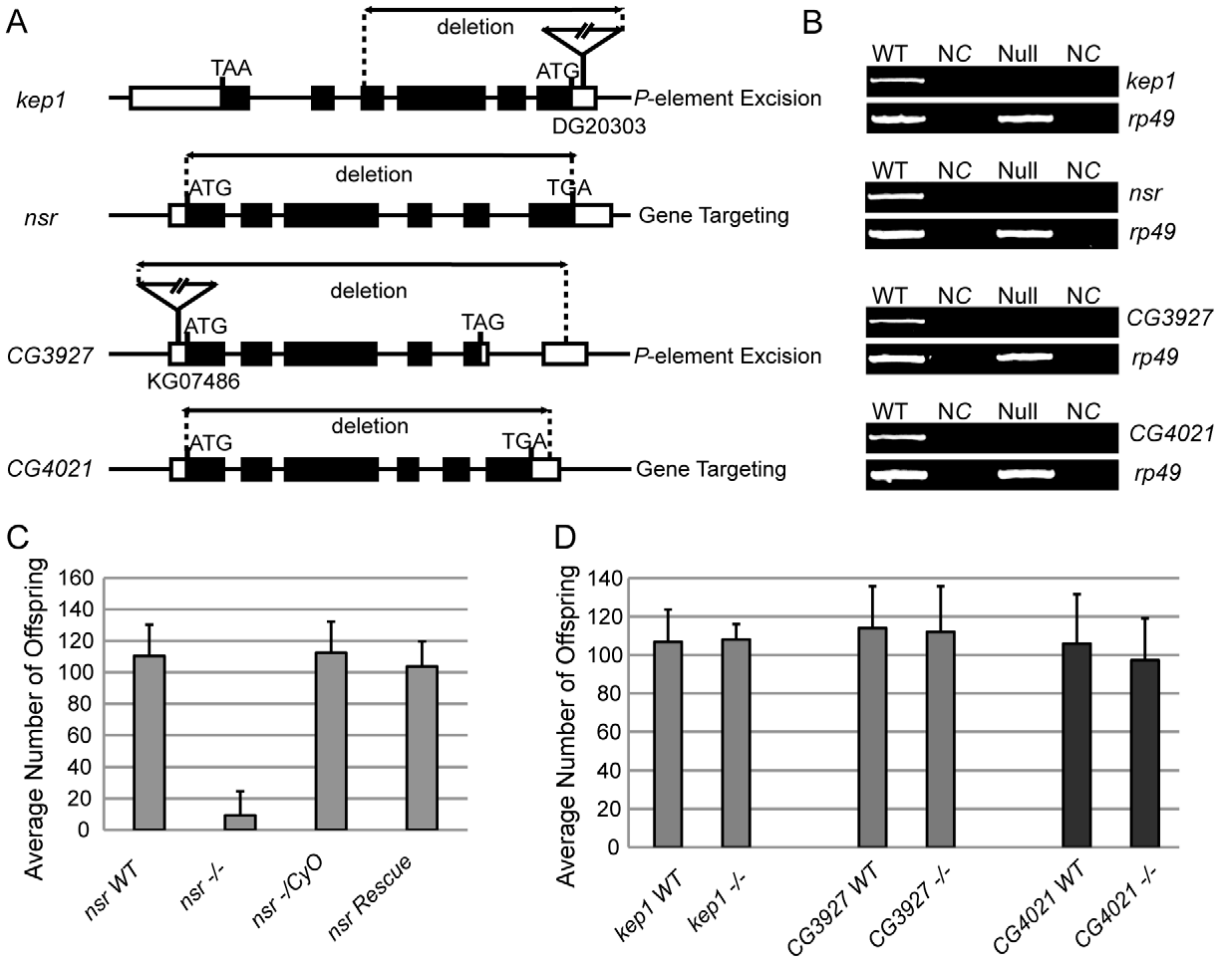
Generation of null mutants for each *kep1* family gene and the male fertility test. (A) Schematic maps of mutant alleles of *kep1* family genes generated by *P*-element excision or targeted mutagenesis. The exons (black block), start codon (ATG), stop codon (TAA/TGA/TAG), and deleted genomic regions are indicated. For *kep1* and *CG4021*, the *P*-element insertion for further excision is shown by a triangle, with a Bloomington stock number given underneath. (B) RT-PCR examination of null mutants (Null) for each *kep1* family gene relative to WT flies (WT). Negative control (NC) is the reaction without reverse transcriptase, and the expression of *rp49* is used as internal control. (C) Fertility test for *nsr* WT and mutant males. *nsr WT*: WT controls with identical genetic background with *nsr* mutants; *nsr -/-*: homozygous *nsr* mutants; *nsr -/CyO*: heterozygous *nsr* mutants; *nsr Rescue*: flies with a copy of WT *nsr* transgene in the *nsr* mutant background. Error bars indicate standard deviation. (D) Fertility test for *kep1*, *CG3927,* and *CG4021* WT and mutant (-/-) males. Error bars indicate standard deviation.

**Figure 4:**
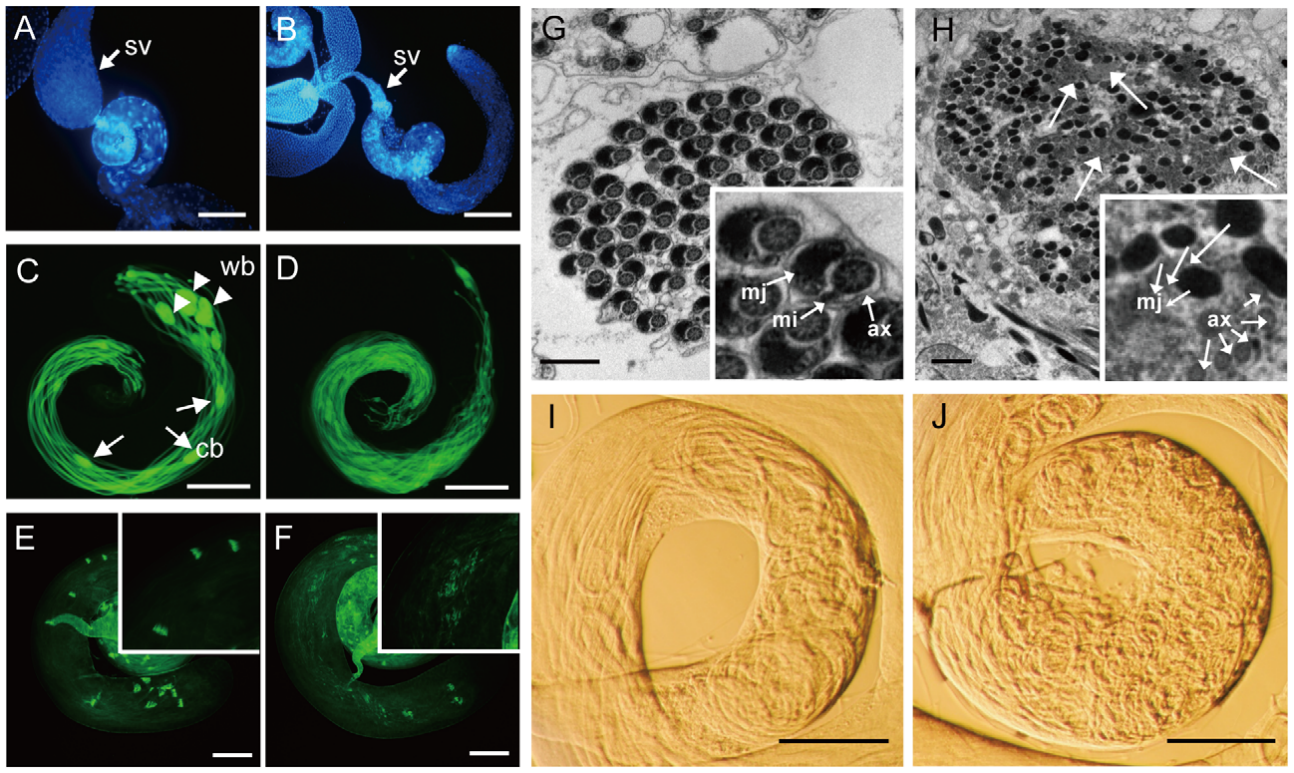
Morphological characterization of *nsr* mutants. (A and B) Seminal vesicles (sv) from *nsr* WT (A) and mutant males (B) stained by Hoechst 33342. A shriveled seminal vesicle was observed in *nsr* mutants. (C and D) *dj*-GFP labeled elongated spermatids from WT and *nsr* mutant testes. The elongated spermatids from WT testis (C) are tightly organized in bundles with visible cystic bulges (cb) (arrowhead) and waste bags (wb) (arrow). In contrast, the elongated spermatids from *nsr* mutants (D) are much looser; the structures of cb and wb can hardly be observed. (E and F) Phalloidin-stained ICs in testes of WT and *nsr* mutants (inset shows a region at higher magnification). In WT testis, the ICs progress syncytially (E), while the syncytial movement of ICs is abnormal in *nsr* mutant testis (F). (G and H) Electron microscopic images of cyst from WT and *nsr* mutant testes at the late stage of individualization (inset shows a region at higher magnification). Individualized spermatids in WT cyst contain highly ordered organelles, including the major mitochondrial derivative (mj), the minor mitochondrial derivative (mi), and the axoneme (ax) (G), while the spermatid individualization is abolished in the *nsr* mutant cyst, as revealed by excess cytoplasmic remnants (arrow) and poorly assembled organelles (H). (I and J) Testis bases of WT and *nsr* mutant flies under a phase contrast microscope. Unlike regular coiling in the WT flies (I), the spermatids are tangled at the testis base of *nsr* mutant (J). Scale bars: 200 µm for A–D; 100 µm for E and F; 1 µm for G and H.

To comprehensively understand the biological functions of the *kep1* family genes, we have generated null mutants for all four intact gene copies in *D. melanogaster* by either gene targeting knockout [27] or imprecise *P*-element excision [28] (Figure 3A and 3B). The wild-type (WT) control flies of the mutants are WT recombinants created by targeted mutagenesis or precisely excised strains of *P*-element excision, for the sake of an identical genetic background between the mutant and the WT flies. The null males of *nsr* display significantly reduced fecundity when compared to the WT males (*p*-value <0.001, Mann-Whitney U test) (Figure 3C). This phenotype can be fully restored by introducing the genomic sequences of *nsr* back into the genome (Figure 3C). Heterozygous flies of *nsr* mutants are equally fertile as the WT flies (Figure 3C). We found that the sperm storage tissue (seminal vesicle) of *nsr* male mutants was empty or contained little sperm, if any (Figure 4A and 4B). During *D. melanogaster* spermatogenesis, germ cells from gonial precursors differentiate into cysts of 64 syncytial spermatids, which will undergo an actin-based individualization process, in which a bulk of unneeded cytoplasm is eliminated from the spermatids through remodeling of the cyst membrane. Extrusion of the cytoplasm along sperm bundles can form visible cystic bulges, which will migrate to the distal ends and are detached as waste bags. An actin structure, termed the “investment cone (IC),” is formed at the site where each spermatid develops its own membrane [29], [30]. We labeled the sperm bundles together with the cystic bulges and waste bags with GFP under control of the *don juan* (*dj*) gene promoter [31], and the ICs are visualized by FITC-conjugated phalloidin. The testis of *nsr* mutant male contains comparable amounts of spermatids as their WT controls; however, the structures of cystic bulges and waste bags are largely absent (Figure 4C and 4D). In WT flies, ICs in the same cyst move coordinately in clusters (Figure 4E), while they are scattered along the sperm bundles in the *nsr* mutants (Figure 4F). The phenotypes above are typical features of an impaired individualization process [30]. Electron microscopy examination further confirmed that the spermatids of *nsr* mutants are unindividualized, with substantial amounts of residual cytoplasm (Figure 4G and 4H). As the final step of spermatogenesis, the spermatids are assembled by coiling at the base of the testis to facilitate their transport into seminal vesicles [29]. Under a phase-contrast microscope, the sperm bundles of *nsr* mutants are twisted at the distal ends of testis, instead of regular coiling (Figure 4I and 4J). Therefore, *nsr* is functionally involved in both sperm individualization and coiling.

In contrast, though *kep1* is required for female fertility in *D. melanogaster* [16], no significant difference in male fertility was detected between *kep1* mutant males and their WT controls (Figure 3D). Also, we did not observe reduced fertility (Figure 3D) or other obvious defects for the *CG3927* and *CG4021* mutants. Considering that only *nsr* exhibits a robust signature of positive selection, this result may not be surprising. Either *CG3927* and *CG4021* have not acquired new functions or their phenotypic effects are not strong enough to be detected in our phenotyping assay.

### Requirement of *nsr* for the Integrity of Sperm Axoneme Structure by Regulating Several Y-Linked Male Fertility Genes

Microarray comparison of the transcription profiles between *nsr* WT and mutant testes only identified 14 genes that exhibited at least a 2-fold difference at the expression level, but none of them seemed to be male fertility-related (Table S3). Considering that the background hybridization noise and lack of probes for some genes might limit the power of microarray, we further implemented whole transcriptome shortgun sequencing (RNA-Seq), which is regarded as a more precise way for measurements of transcript levels [32]. Using the Illumina paired-end sequencing platform, we generated 16.3 million reads (75-bp) for WT testes and 9.6 million for *nsr* mutant testes. Based on these transcriptome data, we identified 10 genes that were significantly differentially expressed (>5-fold) between WT and mutants. Among them, *kl-2*, *kl-3,* and *kl-5* are known male fertility genes, and the others are either not correlated with male fertility or functionally unknown (Table S4).

**Figure 5:**
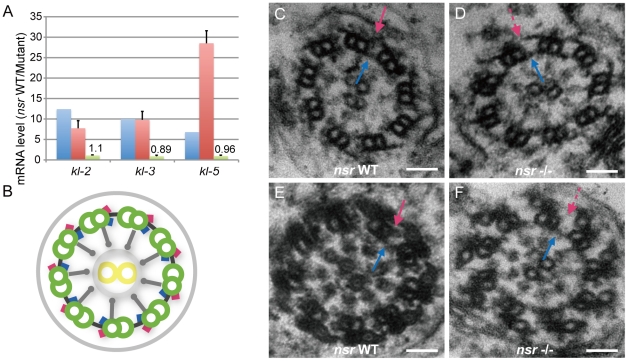
Identification of the Y chromosome genes *kl-2*, *kl-3,* and *kl-5* as downstream targets of *nsr*. (A) Histogram of *kl-2*, *kl-3,* and *kl-5* RNA level changes in testes of *nsr* mutants for mature transcripts, estimated by RNA-Seq (blue) and quantitative real-time PCR (red), and for primary transcripts estimated by quantitative real-time PCR (green). Error bars indicate standard deviation. Both RNA-Seq and real-time PCR results show that the mature transcripts are down-regulated in the mutant testes. The difference between the estimations of *kl-5* by RNA-Seq and real-time PCR is possibly due to the larger variations of genes with lower abundance in RNA-Seq [62]. The real-time PCR result shows that the levels of primary transcripts are largely unaltered between the WT and mutant testes. (B) Graphic illustration of axoneme structure [63], [64]. The axoneme is composed of a central pair of singlet microtubules (yellow circle) surrounded by nine doublet microtubules (green circle), anchored by outer (red) and inner (blue) dynein arms that can mediate axoneme motility. Radical spokes (grey spoke) pass from each doublet fiber toward the central singlets. (C–F) Electron microscopic images of early- (C and D) and late-stage (E and F) spermatid axonemes for WT and *nsr* mutants. The late-stage axoneme is distinct from early-stage axoneme by extensive accessory structures. For both stages, the inner dynein arms of the axoneme are normal in *nsr* mutants (blue arrow), but the outer dynein arms (red arrow) are constantly missing (dashed red arrow). Scale bars: 50 nm.

The *kl-2*, *kl-3,* and *kl-5* genes are 12.4-fold, 10.0-fold, and 6.84-fold down-regulated in the mutants, respectively (Table S4), and their sharp reductions in expression were validated by real-time PCR (Figure 5A). Interestingly, the three genes were located adjacently on the Y chromosome, and all encode dynein heavy chain polypeptides of the sperm axoneme [33]–[35]. The phenotypic defect associated with the sterility of *kl-2* mutants is not very clear [36], [37], while *kl-3* or *kl-5* mutations by *P*-element insertions result in loss of the outer dynein arm of the sperm axoneme and irregular coiling of spermatid tails, and complete deletion of either locus causes defects in sperm individualization [37]–[39]. Electron microscope examination of the spermatid flagellum showed that the outer dynein arms of sperm axonemes were also missing in the *nsr* mutants (Figure 5B–5F). The deficiencies of *nsr* mutants, including sperm individualization, coiling, and axonemal structures, fit well with the phenotypes of the *kl-3* and *kl-5* mutants. This substantial agreement of the loss-of-function phenotypes between the Y-linked genes *kl-3*, *kl-5,* and *nsr* indicates that *nsr* is involved in male functions by regulating *kl-3*, *kl-5,* and, possibly, *kl-2* as well. Moreover, it is very likely that *nsr* regulates the *kl-2*, *kl-3,* and *kl-5* genes post-transcriptionally, because their primary transcript levels are largely unaltered between the mutants and WT flies, as shown by real-time PCR results (Figure 5A). This is also in accordance with the conserved RNA-binding domain (Figure 1B and 1C) and the splicing factor-like distribution pattern (Figure 2F) of the Nsr protein. More importantly, our co-immunoprecipitation experiment demonstrated that the pre-mRNA cleavage stimulatory factor CstF-64 [40] can be specifically immunoprecipitated by TAP-tagged Nsr protein from testis extracts (Figure S3A and S3B). This result fortifies the idea that *nsr* might function as an RNA processing factor, although future studies are needed to explore how *nsr* and *CstF-64* collaboratively process the primary transcripts of these male genes.

### Functional Status of Ancestral *kep1* in the Pre-Duplication Species *D. yakuba*

**Figure 6:**
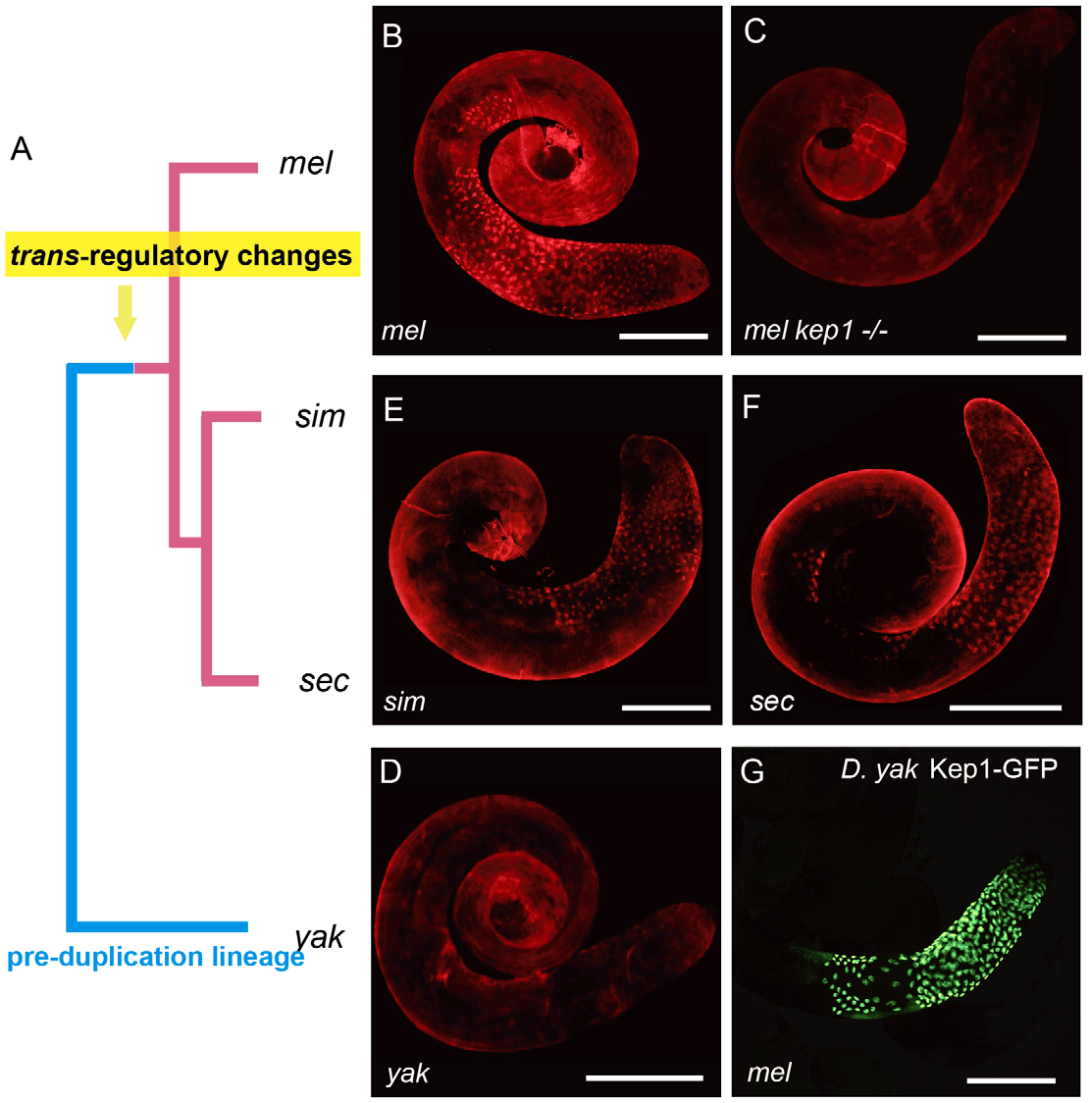
Evolutionary history of the expression patterns of *kep1* family proteins. (A) Phylogenetic tree of *Drosophila* species. The pre- and post-duplication lineages are denoted by blue lines and red lines, respectively. (B–F) Immunostaining of testes with different genotypes using Kep1 antibody shows fluorescent signals in primary spermatocytes for the *D. melanogaster* species complex (B, E and F) but only background staining for the pre-duplication species *D. yakuba* (D). This suggests that the primary spermatocyte-biased expression patterns of *kep1* family genes should have been established in the common ancestor of the *D. melanogaster* species complex after the split of *D. yakuba*. (G) Transgenic GFP, regulated by *cis*-elements (including promoter, 5′ UTR and coding sequences) of *D. yakuba kep1* in *D. melanogaster,* is also enriched in primary spermatocytes, indicating that the *cis*-elements of *kep1* have not changed between *D. melanogaster* and *D. yakuba*, and thus, *trans*-regulatory changes should have contributed to the observed testicular expression patterns of *kep1* family genes in *D. melanogaster*. The abbreviations of *Drosophila* species are the same as in Figure 1. Scale bars: 200 µm.

We traced the functional status of *kep1* in the pre-duplication species *D. yakuba* by detecting its expression pattern using Kep1 antibody (Figure S3C). Surprisingly, immunocytochemistry with Kep1 antibody showed only background staining of *D. yakuba* testis (Figure 6D), whereas it is capable of yielding a robust staining pattern in the primary spermatocytes of *D. melanogaster* (Figure 6B and 6C), exactly as revealed by transgenic GFP localization (Figure 2A). The antibody worked well in detecting Kep1 proteins in ovary extracts from both *D. yakuba* and *D. melanogaster* by Western blot (Figure S3D), ruling out the possibility that the antibody sensitivity is not equally sufficient for detecting Kep1 protein of *D. yakuba*.

Absence of Kep1 protein in *D. yakuba* testis suggests that the *kep1* gene should not be ancestrally required for male fertility, and it also raises the questions of when and how the novel testicular expression patterns of the *kep1* family in *D. melanogaster* has been evolved. The immunofluorescent signals of Kep1 proteins in the sibling species of *D. melanogaster*, *D. simulans* (Figure 6E) and *D. sechellia* (Figure 6F), suggest that this novel pattern has been established in the common ancestor of the *D. melanogaster* species complex. This interspecies difference of expression pattern between *D. yakuba* and *D. melanogaster* may arise from either *cis*-acting or *trans*-acting regulatory changes. The two genetic factors can be distinguished by testing the transcriptional activity of *D. yakuba*'s *cis*-elements of *kep1* in *D. melanogaster*. Controlled by *D. yakuba*'s *cis*-elements of *kep1*, GFP was also found to accumulate in the primary spermatocytes in *D. melanogaster* (Figure 6G) with the same subcellular localization as with the control of the *cis*-elements of *D. melanogaster kep1* (Figure S3E). This means that the activity of the *cis*-elements has not been differentiated between *D. yakuba* and *D. melanogaster*, and it is the changes in *trans*-regulatory repertoires that most likely have enabled all *kep1* family genes to obtain novel testicular expression patterns.

## Discussion

There are two possible scenarios to explain the current functional roles of *nsr* in *D. melanogaster*: neofunctionalization and subfunctionalization [4], [5]. Our results tend to support the neofunctionalization scenario, although we cannot completely exclude the possibility of subfunctionalization.

Several pieces of evidence support the neofunctionalization scenario. Firstly, the parental gene *kep1* is under strict purifying selection across the *Drosophila* phylogeny (Table S1). The significant conservation of *kep1* and its inessentiality for male fertility in the pre-duplication species *D. yakuba* is consistent with the reported functions of *kep1* in female fertility, eye development, and immune response [16] but not male fertility (Figure 3D) in *D. melanogaster*. These results suggest that *kep1* possibly has retained its ancestral functions without evolving novel male functions after the duplication events, and *nsr* is free to evolve new functions. Secondly, *nsr* shows a robust signal of positive selection (Figure 1C and 1D), especially in the C-termini (Figure 1C). As we know, RNA recognition is a complex biological process that may need the collaboration of multiple factors; the RNA-binding domain alone possibly does not contain sufficient information for specific targeting [41], [42]. Thus, the rapidly evolving C-termini of *nsr* could have contributed to novel RNA-binding ability by mediating co-option with different cofactors, and this idea is further strengthened by the specific immunoprecipitation of the pre-mRNA cleavage stimulatory factor CstF-64 by the Nsr protein (Figure S3B). The subcellular localization pattern of the Nsr protein is also different from the Kep1 protein by displaying a larger localization range in the nuclei of primary spermatocytes (Figure 2I), and cell type-specific expression or subcellular localization is regarded as one of the strategies for RNA-binding proteins to regulate specific splicing events [42]. Although it is still not clear what is the concrete molecular process that the novel distribution pattern of *nsr* has contributed to its roles in spermatogenesis, it is possible that this novel distribution might allow the spatial-specific assembling between *nsr* and its cofactors, and the subsequent specific regulation of mRNA substrates. Thirdly, our antibody did not detect obvious expression of Kep1 protein in *D. yakuba* testis, and thus, the parental gene *kep1* should not be ancestrally required for male fertility. After the split of *D. yakuba*, *trans*-regulatory changes possibly occurred prior to or accompanied by the duplications of *kep1*, which enabled the *kep1* family genes to obtain novel testicular expression patterns and thereby lend them an opportunity to evolve novel male functions, as *nsr* has done.

Nevertheless, the alternative subfunctionalization scenario cannot be completely excluded if a recent “gain and loss” turnover of male functions for *kep1* did happen or if *kep1* has lost its male functions in the *D. yakuba* lineage for some reason. In the recent “gain and loss” turnover, the parental gene *kep1* could have acquired an essential role in spermatogenesis after the split of *D. melanogaster* and *D. yakuba* but prior to the duplication events, whereas the new copy *nsr* has taken over the spermatogenesis role from *kep1* after its origination.

The new duplicate gene *nsr* displays tremendous divergence from *kep1* at the levels of biological function and molecular pathway. The *kep1* gene participates in female fertility by regulating the apoptosis molecule *dredd* [16], whereas the new gene *nsr* is integrated into the spermatogenesis pathway by regulating Y-linked male fertility genes; thus, our findings also provide an unusual case, showing a functional transition in a new gene from a female role to male role. It is interesting that the newly originated genes are often expressed primarily in male reproductive tissues in diverse organisms [43]–[47], and most of the new *Drosophila* genes with known functions [6]–[8], together with *nsr*, are associated with male reproduction. This phenomenon pronounces that new genes may tend to be functionally male-biased and suggests a significant role of natural selection and sexual selection in the fixation of beneficial mutations for male reproductive success.

Our study reveals that *nsr* has been integrated into fundamental developmental processes by regulating pre-existing essential genes. Interestingly, the sperm maturation aspects that *nsr* participates in are conserved during evolution [48]. For example, the failure of eliminating sperm cytoplasm and loss of the outer axonemal dynein arm can also cause many types of human infertility [49]–[51]. The functional mechanism of *nsr* indicates that new genes could contribute to the evolutionary turnover of molecular pathways governing essential and conserved developmental processes, which partially explains the phenomenon that the same developmental processes in different organisms are sometimes achieved by a different set of genes. The positive selection signal and biological functions of *nsr* together strongly suggest that *nsr* might have contributed to the adaptive evolution of male reproductive pathways in the *D. melanogaster* species complex.

## Materials and Methods

### Evolutionary Analysis

Protein sequences of *nsr*, *CG3927*, *CG4021,* and *kep1* in *D. melanogaster* are downloaded from FlyBase (http://flybase.org) and aligned by ClustalW (http://www.ebi.ac.uk/Tools/clustalw). Orthologous coding sequences of *kep1* family genes in other *Drosophila* species (http://flybase.org) were predicted using a combination of BLAT (http://genome.ucsc.edu) and GeneWise (http://www.ebi.ac.uk/Tools/Wise2) and manually checked. Alignments of coding sequences mentioned below are performed by MEGA 3.2 [52], considering the coding structures. To estimate the selective constraint on *kep1* through the *Drosophila* phylogeny, alignments of *kep1* coding sequences from different *Drosophila* species were tested for purifying selection by MEGA 3.2 pairwisely. To detect the selective pressure on the new genes of the *kep1* family, alignments of the coding sequences between *kep1* and each new gene were performed and calculated for the *dN*/*dS* ratio with 120-bp windows and 6-bp slides. For each window, the maximum likelihood method [53] was used to test if the *dN*/*dS* ratio was significantly different from one (two-tailed Fisher's exact test).

The *ω* (*dN*/*dS*) values in the phylogeny of new *kep1* family genes were estimated using the maximum likelihood approach, implemented by the codeml free-ratio model in the PAML4.2 package (http://abacus.gene.ucl.ac.uk/software/paml.html) [17]. To test if the *ω* ratio in the ancestral lineage of the *D. melanogaster* species complex was significantly different from one, the likelihood of the two-ratio model with an estimated *ω* was compared to an alternative two-ratio model, with *ω* constrained to be one for this lineage.

### Fly Strains

All *Drosophila* strains were maintained at 25°C using standard cornmeal medium. The transgenic strains were produced by microinjection of *w1118* embryos following standard *P*-element-mediated germline transformation [54]. *P*-element insertion stocks DG20303 and KG07486 were ordered from Bloomington Stock Center. Strains for *P*-element excision (*Sp*/*CyO; Δ2-3, Sb*/*TM6B* and *Sp*/*CyO; MKRS*/*TM6B*) are kindly provided by Dr. Yongqing Zhang. Strains for targeted mutagenesis (*70FLP70I-SceI*, *70FLP* and *70I-CreI*) were previously described by Xie and Golic (2004).

### Transgenic Constructs of *kep1* Family Genes

For GFP-tagged vectors, the pH-Stinger plasmid [55] was modified by excision with *SpeI*/*NheI* and re-ligation to remove its Hsp70 promoter and nuclear GFP. Gene promoter sequences (plus 5′ UTR) and GFP coding sequences were then cloned into *XbaI*/*EcoRI* and *EcoRI*/*KpnI* sites of the modified plasmid. Coding sequences of each gene were added into *EcoRI* sites and selected for correct insertion orientation (Figure S2B). TAP-tagged transgenic vectors were constructed similarly but had GFP replaced with a TAP tag, which consists of two IgG-binding domains of protein A (ProtA) and a calmodulin-binding peptide (CBP) separated by a TEV protease cleavage site [56] (Figure S2C). For all the vectors above, a homologous upstream region of *kep1* family genes (including *D. yakuba kep1*) was adopted as the promoter sequence (Figure S2D). A rescue construct of *nsr* was prepared by inserting a 2.8-kb DNA fragment, ranging from the end of the upstream gene to the start of the downstream gene, into the *NotI* site of the pW8 transformation vector (FlyBase). The primer information is available in Table S5.

### Generation of Null Flies of Each *kep1* Family Gene and Male Fertility Test

*P*-element excision: The fly strains DG20303 (with a *P*-element at the 5′ UTR of *kep1*) and KG07486 (with a *P*-element annotated to locate at the *nsr* locus but found to be inserted at the 5′ UTR of *CG3927* after PCR validation) were mobilized with *Δ2-3* transposase by standard *P*-element excision, respectively [28]. Excision lines were screened by PCR, and the endpoints were determined by sequencing.

Gene knockout by ends-in targeting: The targeting vectors were designed to create a deletion, spanning from 42-bp downstream of the transcriptional start site to a site within the 3′ UTR of *nsr,* and a deletion spanning from the start codon to a site within the 3′ UTR of *CG4021*, respectively. Targeted mutagenesis was performed as previously described [27]. Donor flies bearing the targeting vector were generated and crossed with flies carrying heat shock-activated FLP recombinase and I-SceI endonuclease (*70FLP70I-SceI*). The 0-3 day old progeny were heat-shocked at 38.5°C for 1 hour, and the enclosed white-eye virgins were crossed with males constitutively expressing FLP recombinase (*70FLP*). In total, at least 1000 vials were screened for nonmosaic red-eye individuals with successful insertions of the targeted allele at the site of the endogenous allele. Stocks of the recombinant flies were established and crossed with flies carrying heat shock-activated I-CreI endonuclease (*70I-CreI*). We heat-shocked 0-3 day old progeny at 38.5°C for 1 hour and screened for white-eye adults with recombinant reduction events at the targeted site. The reduction events will lead to either removal of the allele or maintenance of the WT allele. Strains of both genotypes were established to serve as knockout and WT lines, respectively.

For the male fertility test, an individual male of each genotype (<1 day) was placed with three *w1118* virgin females, which were collected within 5 hours of enclosure and aged for 2 days. The progeny were counted on the 18th day after the mating and compared between the mutant and WT lines using Mann-Whitney U test.

### Antibody Preparation and Immunofluorescence Assay

A polyclonal antibody was raised against the glutathione-S-transferase-Kep1 (amino acids 233–313) recombinant protein in guinea pigs. Testis squashes and immunostaining were performed as previously described [57]. The primary antibodies used are guinea pig anti-Kep1 serum (1∶200 dilution) for Kep1 protein and rabbit peroxidase-antiperoxidase complex (PAP) (1∶1000 dilution, Sigma) for ProtA. The secondary antibodies are Alexa 555-conjugated anti-guinea pig and Alexa 594-conjugated anti-rabbit (Molecular Probes). Testes were co-stained with Hoechst 33342 (1 µg/ml, Molecular Probes) to visualize nuclear DNA if needed. FITC-conjugated phalloidin (1∶100 dilution) was used for IC staining. RNase A treatment was performed as previously described [58] by a 10-min incubation of TBS with 50 µg/ml RNase A (Fermentas), and the controls were incubated in the same buffer, but free of RNase A.

### Western Analysis

For sample preparation, adult testes or ovaries from 0–5 day old flies were dissected in PBS, transferred to RIPA buffer, ground, and boiled at 95°C for 10 min for lysis. The primary antibodies used were PAP (1∶2000 dilution, Sigma), mouse anti-β-actin (1∶3000 dilution, Abcam), and guinea pig anti-Kep1 (1∶500 dilution). Peroxidase-conjugated secondary antibodies were used for signal detection (1∶10000 dilution, Santa Cruz).

### Immunoprecipitation Assay

Six hundred testes of 0–3 day old flies carrying TAP-tagged Kep1 protein, TAP-tagged Nsr protein, or TAP-tagged CG4021 protein were used for affinity purification, respectively. Testes were ground in 100 µl RIPA buffer plus protease inhibitor cocktail (Roche) with the Sample Grinding Kit (GE Healthcare). The cell suspension was centrifuged at 4°C for 5 min, the supernatant was pre-cleared by 5 µl protein G plus-agarose beads (Santa Cruz), and incubated with 2 µl PAP at 4°C overnight. Then, 10 µl protein G plus-agarose beads were added to the mixture and incubated at 4°C for 1 hour. Complexes of TAP-tagged proteins were liberated from the beads by cleavage of TEV protease as previously described [56], subjected to SDS-PAGE, and visualized by Coomassie blue staining. The protein band of interest was cut out and identified by MALDI-ToF mass spectrometry.

### Electronic Microscopy

The dissected testes from WT controls and *nsr* mutants were fixed in 2.5% glutaraldehyde, washed twice with PBS, post-fixed with OsO4, and dehydrated in an ascending series of ethanol. The resultant specimens were embedded in Araldite, sliced into ultrathin sections (50–100 nm), stained with 1% uranyl acetate, and examined with a JEOL electron microscope.

### RT-PCR and Real-Time RT-PCR

Total RNA was isolated from adult testes with Trizol reagent (Invitrogen) and treated with DNase I (Fermentas). Reverse-transcription was performed using the RevertAid First Strand cDNA Synthesis kit (Fermentas) with a no-reverse-transcriptase reaction as the negative control. Real-time PCR was performed in triplicate with SYBR Green PCR Mix (Bio-Rad) and subjected to the ABI 7000 Sequence Detection System. Oligo-dT primer was used to synthesize the cDNA templates for detecting mature transcripts and random hexamer primer for primary transcripts. Information on the PCR primers is available in Table S5. The relative concentration of genes was calculated by analyzing their dissociation curves using the constitutively expressed gene *rp49* as the internal control.

### Microarray Analysis

With Trizol reagent (Invirtrogen), total RNA was extracted from testes of 0–2 day old *nsr* mutant and WT flies, respectively. After amplification, mRNA was fluorescently labeled by GeneChip One-Cycle Target Labeling (Affymetrix) and subjected to GeneChip *Drosophila* Genome 2.0 Arrays (Affymetrix) in duplicate. Image collection was performed in accordance with standard Affymetrix protocols. The significance of gene expression change was estimated using the Significance Analysis of Microarrays (SAM) algorithm, which assigns a score to each gene on the basis of change in gene expression relative to the standard deviation of repeated measurements [59]. The microarray data have been deposited in Gene Expression Omnibus (GEO) (http://www.ncbi.nlm.nih.gov/geo) under accession number GSE22289.

### Paired-End cDNA Library Construction for Illumina Genome Analyzer 2 (GA2) Sequencing

With Trizol reagent (Invirtrogen), 5 µg total RNA was extracted from testes of 0-1 day old *nsr* mutant and WT flies, respectively. The first-strand cDNA was synthesized with oligo-dT primer by Superscripts II reverse transcriptase (Invitrogen), and second strand cDNA synthesis was followed according to the standard protocol. Then, the double-stranded cDNA was purified with the Qiaquick PCR purification kit (Qiagen) and fragmented with a nebulizer (Invirtrogen), resulting in an average size of 150–250-bp. Overhangs of resultant fragmented cDNAs were blunted with T4 DNA polymerase (NEB) and Klenow polymerase (NEB) and treated with 3′-5′ exonuclease-deficient Klenow polymerase (NEB) to generate 3′ overhangs. After that, cDNA was ligated to an Illumina PE adapter oligo mix by the Quick ligation kit (Qiagen). The adapter-modified cDNA within 200-bp was isolated by agarose gel, extracted with the QIAquick Gel Extraction Kit (NEB), and amplified by PCR reaction. Finally, the library products were sequenced using the Illumina GA2 sequencing machine. Sequence data from this study have been submitted to the NCBI Short Read Archive (http://www.ncbi.nlm.nih.gov/Traces/sra/sra.cgi) under accession number SRA020074.

### Measurement of Gene Expression Using Data of Illumina GA2 Sequencing

The generated 75-bp raw reads were mapped to the genomic sequences of *D. melanogaster* (Ensembl release 55: ftp://ftp.ensembl.org/pub/release-55/fasta/drosophila_melanogaster) using SOAP2 software (http://www.soapmaker.ca) [60]. The count of covering reads for each annotated transcript (Ensembl release 55: ftp://ftp.ensembl.org/pub/release-55/gtf/drosophila_melanogaster) was calculated as the index of their expression level. The alteration of transcript level between *nsr* mutants and WT flies was estimated and normalized for the variation of the total data size of transcript reads. The significance of expression difference (*p*-value) for each gene (the longest transcript) was further computed according to the R package “DEGseq” using the MA-plot-based method with a random sampling model and followed by an adjustment with *q*-values for multiple testing corrections [61].

## Supporting Information

Figure S1 Evolutionary analysis of *CG3927* and *CG4021*. (A) Distributions of *dN/dS* throughout *kep1-CG3927* and *kep1-CG4021* pairs. With 120-bp windows and 6-bp slides, *dN/dS* ratios were estimated using the maximum likelihood method and plotted. Blue spots represent *dN/dS* ratios that are statistically significantly lower than the neutral expectation (p-value <0.05, two-tailed Fisher's exact test). Regions embedded in the KH domain, as depicted by the grey block, are enriched with signals of purifying selection. The shorter alignment of the *kep1-CG3927* pair than the *kep1-nsr* and *kep1-CG4021* pairs is mostly due to less well-aligned sequences caused by indels. (B) Likelihood values of nucleotide substitutions for *CG3927* and *CG4021* in *Drosophila* lineages. Numbers of nonsynonymous and synonymous substitutions for the entire coding sequences are labeled above the lineages, and ω values (*dN/dS*) are labeled beneath the lineages. Abbreviations: *D. melanogaster* (*mel*); *D. simulans* (*sim*); *D. sechellia* (*sec*); *D. yakuba* (*yak*), and *D. erecta* (*ere*).
(0.48 MB TIF)

Figure S2 Expression analysis of *kep1* family proteins. (A) Male-specific expression pattern of new *kep1* family genes shown by RT-PCR. Total RNA was extracted from the whole body of 0–5 day old adults. F: female; M: male. Negative control (NC) was reaction without reverse transcriptase. The expression of *rp49* was used as the internal control. (B and C) Schematic representations of GFP (B) and TAP (C) transgene constructs. CDS: coding sequences. (D) Alignments of the homologous promoter sequences of *D. melanogaster kep1* family genes and *D. yakuba kep1*. The asterisks denote the position of identical nucleotides. (E–H) The localization sensitivity of GFP-tagged Nsr protein to RNase A treatment. The localization of GFP-tagged Nsr protein exhibits ectopic diffusion and accumulation after RNase A treatment (E, G) compared with the mock-treated control (F, H). Scale bars: 200 µm for E and F; 20 µm for G and H.
(1.42 MB TIF)

Figure S3 Immunoprecipitation results of TAP-tagged *kep1* family proteins and pre-duplication ancestral subcellular localization of Kep1 protein. (A) Western blot of testis extracts from 0–5 day old flies, with the indicated genotypes. The blot was probed with PAP (to recognize the ProtA of TAP) and β-Actin antibody (loading control). The absence of ProtA in *w1118* lane indicates the specificity of PAP antibody. (B) Immunoprecipitation of TAP-tagged *kep1* family proteins with testis extracts from 0–3 day flies. A 50-72-kDa band corresponding to CstF-64 (identified by mass spectrometry) was immunoprecipitated by TAP-tagged Nsr protein (lane 2) but not in TAP-tagged Kep1 protein (lane 1) or TAP-tagged CG4021 protein (lane 3). (C) Western blot of testis extracts from 0–5 day old WT flies showing that Kep1 antibody recognizes a band of about 43-kDa (arrow), which is consistent with the reported size of Kep1 protein, and that this band is absent in *kep1* mutants. (D) Western blot of testis (lane 1 and 2) and ovary (lane 3 and 4) extracts of 0–5 day old *D. melanogaster* and *D. yakuba* probed with Kep1 antibody. Kep1 protein is detectable in ovary but not testis of *D. yakuba* (arrow). (E) Overlapping subcellular localization between the *D. melanogaster* TAP-tagged Kep1 (*mel*-Kep1-TAP) and the transgenic GFP-tagged Kep1 protein regulated by the *cis*-elements (including promoter, 5′ UTR and coding sequences) of *D. yakuba kep1* (*yak*-Kep1-GFP) in *D. melanogaster*. Scale bars: 20 µm.
(1.23 MB TIF)

Table S1 dN/dS ratios of *kep1* among *Drosophila* species (Kumar method). All the pairwise species comparisons show that the parental gene *kep1* is subjected to significant purifying selection (p-value <0.001, MEGA Z-test, kumar method). Abbreviations: *D. melanogaster* (*mel*); *D. simulans* (*sim*); *D. sechellia* (*sec*); *D. yakuba* (*yak*); *D. erecta* (*ere*) and *D. ananassae* (*ana*); *D. pseudobscura* (*pse*).
(0.03 MB DOC)

Table S2 Maximum likelihood tests of positive selection for new *kep1* family genes. The *dN/dS* ratios were set to be *ω_0_* for the background branches and *ω_1_* for the foreground branch, which is the phylogenetic lineage from *D. yakuba* to the common ancestor of the *D. melanogaster* species complex.
(0.03 MB DOC)

Table S3 List of genes identified by microarray to be >2-fold differentially expressed between testes of *nsr* WT and mutant flies. Significance Analysis of Microarrays (SAM) identified 14 genes that changed at least 2-fold with a *q*-value cutoff of 0.01 for significance.
(0.02 MB XLS)

Table S4 List of genes identified by RNA-seq to be >3-fold differentially expressed between testes of *nsr* WT and mutant flies. The significance of expression difference (*p*-value) for each gene (longest transcript) was computed using MA-plot-based method with a random sampling model and followed by an adjustment with *q*-values for multiple testing corrections. With a *p*-value cutoff of 0.001, 10 genes (red) and 43 genes are identified to be significantly changed at least 5-fold and 3-fold, respectively.
(0.04 MB XLS)

Table S5 Primer Information. F: forward primer; R: reverse primer.
(0.03 MB XLS)
