# Supplementary figures and images for: A Young Drosophila Duplicate Gene Plays Essential Roles in Spermatogenesis by Regulating Several Y-Linked Male Fertility Genes
Source: PLoS Genet. 2010 Dec 23;6(12):e1001255. doi: 10.1371/journal.pgen.1001255 (PMC3009665; doi:10.1371/journal.pgen.1001255)

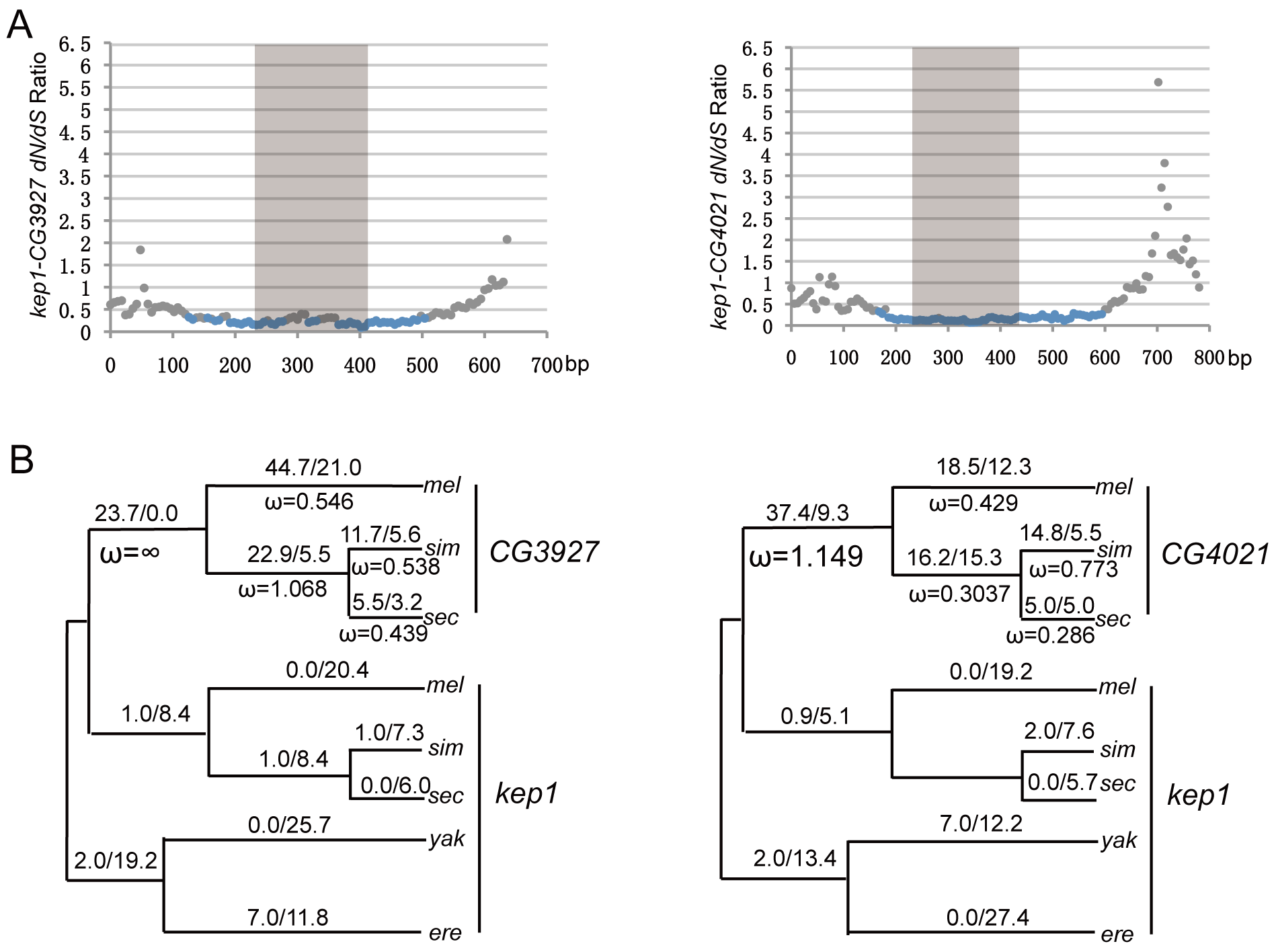

Supplement: Figure S1 — Evolutionary analysis of CG3927 and CG4021. (A) Distributions of dN/dS throughout kep1-CG3927 and kep1-CG4021 pairs. With 120-bp windows and 6-bp slides, dN/dS ratios were estimated using the maximum likelihood method and plotted. Blue spots represent dN/dS ratios that are statistically significantly lower than the neutral expectation (p-value <0.05, two-tailed Fisher's exact test). Regions embedded in the KH domain, as depicted by the grey block, are enriched with signals of purifying selection. The shorter alignment of the kep1-CG3927 pair than the kep1-nsr and kep1-CG4021 pairs is mostly due to less well-aligned sequences caused by indels. (B) Likelihood values of nucleotide substitutions for CG3927 and CG4021 in Drosophila lineages. Numbers of nonsynonymous and synonymous substitutions for the entire coding sequences are labeled above the lineages, and ω values (dN/dS) are labeled beneath the lineages. Abbreviations: D. melanogaster (mel); D. simulans (sim); D. sechellia (sec); D. yakuba (yak), and D. erecta (ere). (0.48 MB TIF) [file pgen.1001255.s001.tif]

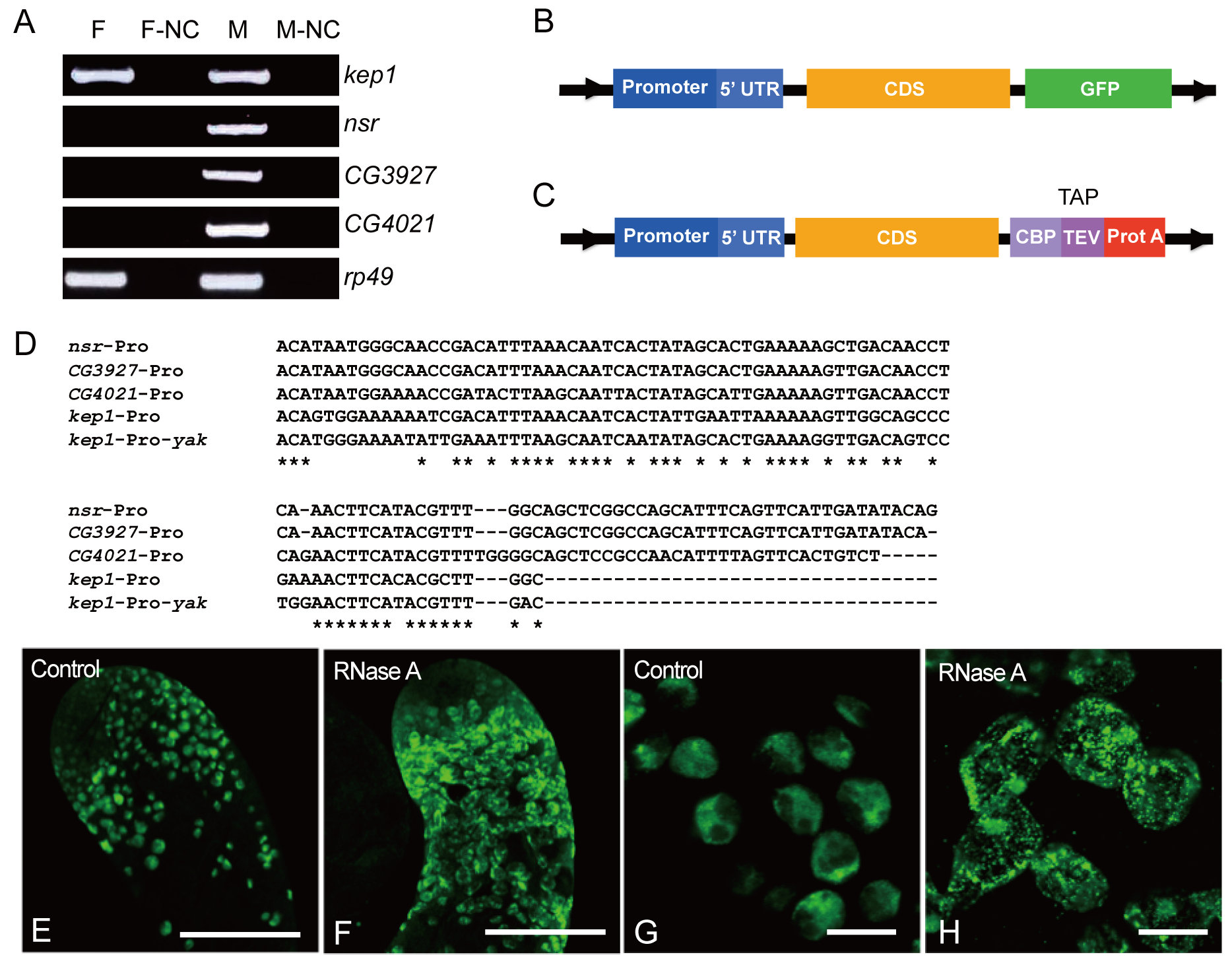

Supplement: Figure S2 — Expression analysis of kep1 family proteins. (A) Male-specific expression pattern of new kep1 family genes shown by RT-PCR. Total RNA was extracted from the whole body of 0–5 day old adults. F: female; M: male. Negative control (NC) was reaction without reverse transcriptase. The expression of rp49 was used as the internal control. (B and C) Schematic representations of GFP (B) and TAP (C) transgene constructs. CDS: coding sequences. (D) Alignments of the homologous promoter sequences of D. melanogaster kep1 family genes and D. yakuba kep1. The asterisks denote the position of identical nucleotides. (E–H) The localization sensitivity of GFP-tagged Nsr protein to RNase A treatment. The localization of GFP-tagged Nsr protein exhibits ectopic diffusion and accumulation after RNase A treatment (E, G) compared with the mock-treated control (F, H). Scale bars: 200 µm for E and F; 20 µm for G and H. (1.42 MB TIF) [file pgen.1001255.s002.tif]

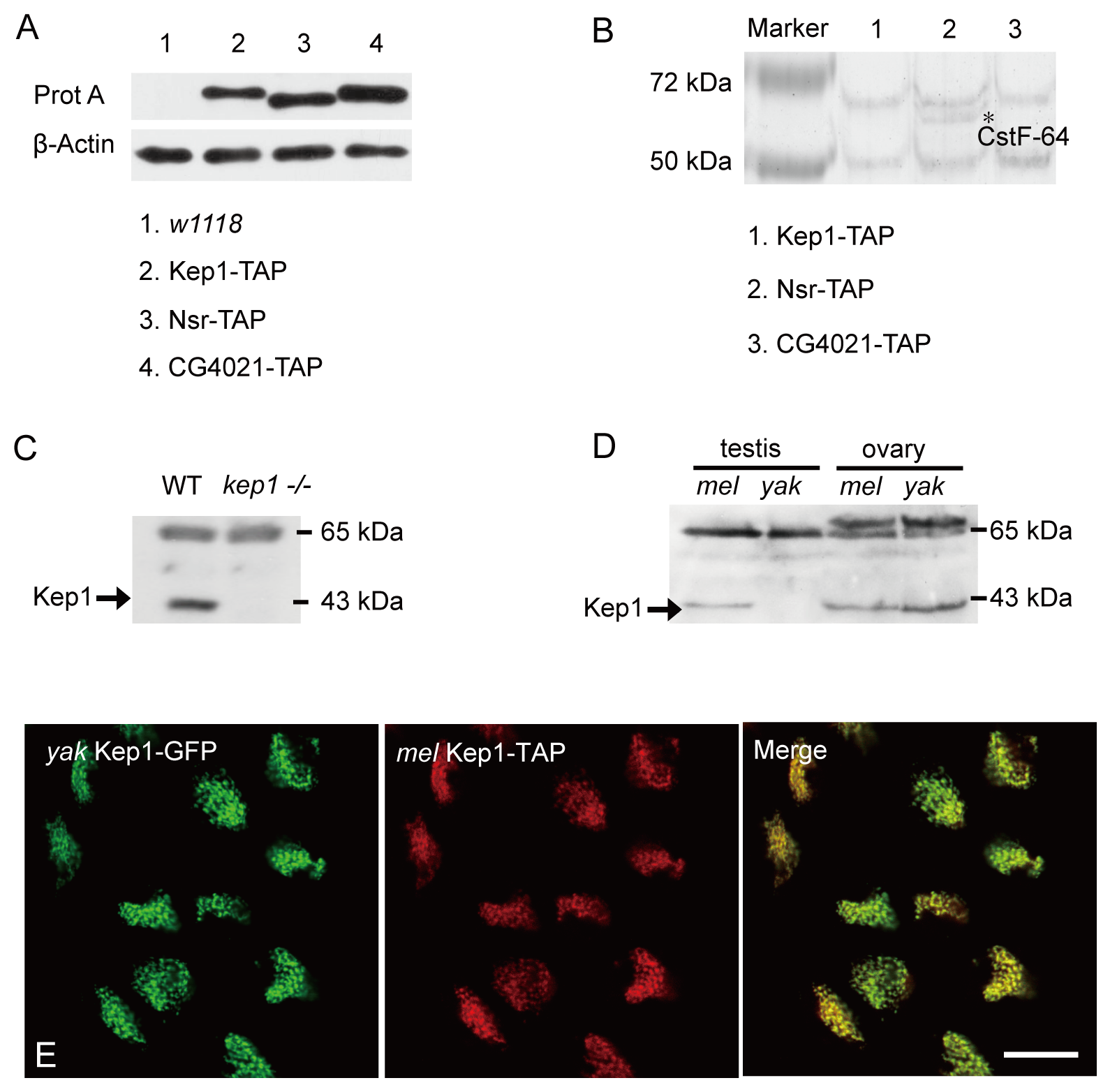

Supplement: Figure S3 — Immunoprecipitation results of TAP-tagged kep1 family proteins and pre-duplication ancestral subcellular localization of Kep1 protein. (A) Western blot of testis extracts from 0–5 day old flies, with the indicated genotypes. The blot was probed with PAP (to recognize the ProtA of TAP) and β-Actin antibody (loading control). The absence of ProtA in w1118 lane indicates the specificity of PAP antibody. (B) Immunoprecipitation of TAP-tagged kep1 family proteins with testis extracts from 0–3 day flies. A 50-72-kDa band corresponding to CstF-64 (identified by mass spectrometry) was immunoprecipitated by TAP-tagged Nsr protein (lane 2) but not in TAP-tagged Kep1 protein (lane 1) or TAP-tagged CG4021 protein (lane 3). (C) Western blot of testis extracts from 0–5 day old WT flies showing that Kep1 antibody recognizes a band of about 43-kDa (arrow), which is consistent with the reported size of Kep1 protein, and that this band is absent in kep1 mutants. (D) Western blot of testis (lane 1 and 2) and ovary (lane 3 and 4) extracts of 0–5 day old D. melanogaster and D. yakuba probed with Kep1 antibody. Kep1 protein is detectable in ovary but not testis of D. yakuba (arrow). (E) Overlapping subcellular localization between the D. melanogaster TAP-tagged Kep1 (mel-Kep1-TAP) and the transgenic GFP-tagged Kep1 protein regulated by the cis-elements (including promoter, 5′ UTR and coding sequences) of D. yakuba kep1 (yak-Kep1-GFP) in D. melanogaster. Scale bars: 20 µm. (1.23 MB TIF) [file pgen.1001255.s003.tif]
