# Supplementary material for: A Young Drosophila Duplicate Gene Plays Essential Roles in Spermatogenesis by Regulating Several Y-Linked Male Fertility Genes
Source: PLoS Genet. 2010 Dec 23;6(12):e1001255. doi: 10.1371/journal.pgen.1001255 (PMC3009665; doi:10.1371/journal.pgen.1001255)
Supplement: Table S1 — dN/dS ratios of kep1 among Drosophila species (Kumar method). All the pairwise species comparisons show that the parental gene kep1 is subjected to significant purifying selection (p-value <0.001, MEGA Z-test, kumar method). Abbreviations: D. melanogaster (mel); D. simulans (sim); D. sechellia (sec); D. yakuba (yak); D. erecta (ere) and D. ananassae (ana); D. pseudobscura (pse). (0.03 MB DOC) [file pgen.1001255.s004.doc]

|  | *mel* | *sim* | *sec* | *yak* | *ere* | *ana* |
| --- | --- | --- | --- | --- | --- | --- |
| *sim* | 0.0338 |  |  |  |  |  |
| *sec* | 0.0140 | 0.0448 |  |  |  |  |
| *yak* | 0.0711 | 0.1009 | 0.0905 |  |  |  |
| *ere* | 0.0244 | 0.0423 | 0.0349 | 0.0745 |  |  |
| *ana* | 0.0418 | 0.0457 | 0.0417 | 0.056 | 0.0456 |  |
| *pse* | 0.0554 | 0.0618 | 0.0588 | 0.0574 | 0.0508 | 0.0505 |
