# Supplementary material for: A Young Drosophila Duplicate Gene Plays Essential Roles in Spermatogenesis by Regulating Several Y-Linked Male Fertility Genes
Source: PLoS Genet. 2010 Dec 23;6(12):e1001255. doi: 10.1371/journal.pgen.1001255 (PMC3009665; doi:10.1371/journal.pgen.1001255)
Supplement: Table S2 — Maximum likelihood tests of positive selection for new kep1 family genes. The dN/dS ratios were set to be ω0 for the background branches and ω1 for the foreground branch, which is the phylogenetic lineage from D. yakuba to the common ancestor of the D. melanogaster species complex. (0.03 MB DOC) [file pgen.1001255.s005.doc]

| Gene | Model (two ratios) | Estimated *ω*_1_ | 2Δl | *p*-value |
| --- | --- | --- | --- | --- |
| *nsr* |  | 7.72 | 4.73 | 0.01 < *p* <0.05 |
| *CG3927* | (*ω_0_*, *ω_1_*=1) vs. (*ω_0_*, *ω_1_*) | ∞ | 9.25 | *p* < 0.01 |
| *CG4021* |  | 1.25 | 0.23 | Not significant |
